# Severe Late‐Onset Vitamin K Deficiency Bleeding Presenting With Subdural Hematoma and Seizures in a 4‐Month‐Old Infant: A Case Report From a Resource‐Limited Setting

**DOI:** 10.1155/crpe/9370038

**Published:** 2026-06-30

**Authors:** Seyed Abdul Ahad Wadoodi, Mohammad Masudi, Ali Rahimi, Abdul Qasim Rahimi, Nasar Ahmad Shayan

**Affiliations:** ^1^ Department of Internal Medicine, Faculty of Medicine, Herat University, Herat, Afghanistan; ^2^ Department of Pediatrics, Faculty of Medicine, Herat University, Herat, Afghanistan; ^3^ Department of Curative Medicine, Faculty of Medicine, Jami University, Herat, Afghanistan; ^4^ Department of Epidemiology and Biostatistics, Western University, London, Ontario, Canada, uwo.ca

**Keywords:** Afghanistan, case report, coagulopathy, infant, late VKDB, seizures, subdural hematoma, vitamin K deficiency bleeding

## Abstract

**Background:**

Vitamin K deficiency bleeding (VKDB) remains a preventable cause of severe hemorrhage in early infancy. Late‐onset VKDB (2 weeks–6 months) most commonly presents with intracranial hemorrhage (ICH) and can result in death or long‐term neurological sequelae. Although routine neonatal intramuscular vitamin K prophylaxis is highly effective, late VKDB may still occur in the presence of contributing factors such as infection or impaired vitamin K availability.

**Case Presentation:**

A 4‐month‐old male infant presented with fever, cough, and poor appetite. He was delivered by cesarean section (birth weight 3200 g) and reportedly received 1‐mg intramuscular vitamin K at birth. He was predominantly formula‐fed with occasional breastfeeding and had no history of trauma or prior bleeding. Initial laboratory testing showed hemoglobin 9 g/dL and leukocytosis; sepsis with anemia was suspected, and intramuscular ceftriaxone was administered. Shortly after the first dose, he developed an expanding injection‐site hematoma with active bleeding. Coagulation studies revealed profound coagulopathy (INR > 4, PT > 120 s, aPTT > 120 s), with a decline in hemoglobin to 6.3 g/dL. Parenteral vitamin K was given, leading to correction of coagulation parameters; however, seizures and deteriorating consciousness developed. Neuroimaging demonstrated a subdural hematoma with hypoxic–ischemic cerebral injury. The patient was managed in the intensive care unit with seizure control, blood transfusion, antimicrobial therapy, and supportive care. Seizures resolved and the neurological status returned to baseline. He was discharged after 10 days without apparent neurological deficits, with long‐term neurodevelopmental follow‐up advised.

**Conclusions:**

This case suggests probable late‐onset VKDB presenting with life‐threatening ICH despite reported neonatal vitamin K prophylaxis, while acknowledging that the complete exclusion of alternative causes of coagulopathy was limited by resource constraints. Clinicians should maintain a high index of suspicion for VKDB in infants with unexpected bleeding after minor procedures or intramuscular injections, particularly in the setting of intercurrent infection, and initiate prompt vitamin K therapy, coagulation assessment, and neuroimaging when indicated.

## 1. Introduction

Vitamin K deficiency bleeding (VKDB), historically known as hemorrhagic disease of the newborn, remains a significant yet preventable cause of infant morbidity and mortality worldwide. Late‐onset VKDB typically occurs between 2 weeks and 6 months of life and is strongly associated with exclusive breastfeeding, absence of vitamin K prophylaxis, and underlying hepatobiliary or malabsorptive disorders [1].

In developing regions where routine neonatal vitamin K prophylaxis is inconsistent, late‐onset VKDB continues to present as life‐threatening intracranial hemorrhages (ICHs), including subdural and subarachnoid bleeds [2, 3]. ICH is reported in 30%–88% of late VKDB cases and carries a mortality rate of up to 20% and a high risk of long‐term neurological sequelae [4].

Case reports from across Asia and Africa highlight that many infants affected by VKDB were exclusively breastfed and did not receive intramuscular vitamin K at birth. For instance, a Japanese infant developed a unilateral subdural hematoma after missing two scheduled prophylactic doses of vitamin K [5]. Similarly, Egyptian studies demonstrated that vitamin K deficiency was implicated in over half of ICH cases in infants, primarily among breastfed babies with diarrhea or recent antibiotic exposure [6, 7].

Despite the proven efficacy of neonatal vitamin K prophylaxis, rare cases have been reported even after a single intramuscular dose at birth. A Serbian series described late VKDB with ICH in exclusively breastfed infants despite intramuscular prophylaxis, suggesting that additional supplementation may be considered in selected high‐risk contexts [8]. In contrast, surveillance data from New Zealand, Australia, and Great Britain indicate that VKDB is now largely concentrated among infants who receive no prophylaxis or incomplete prophylaxis, reinforcing that neonatal vitamin K administration remains essential and highly protective [9–11].

In some cases, late VKDB can mimic nonaccidental injury due to subdural and retinal hemorrhages, which can complicate diagnosis and legal implications [12]. This underlines the importance of considering vitamin K deficiency as a differential diagnosis in infants presenting with unexplained intracranial bleeding.

Given these global trends, late‐onset VKDB remains a preventable yet persistent cause of severe infant morbidity, particularly in resource‐limited settings where follow‐up systems, diagnostic capacity, and parental awareness may be limited [13]. The present case from Herat, Afghanistan, describes a 4‐month‐old infant with clinically probable late‐onset VKDB presenting with injection‐site hematoma, active bleeding, subdural hematoma, and seizures. Its clinical relevance lies in the combination of severe ICH despite reported neonatal prophylaxis and predominant formula feeding, bleeding after intramuscular injection, diagnostic uncertainty in a resource‐limited setting, and favorable apparent neurological recovery despite severe neuroimaging findings.

## 2. Case Presentation

A 4‐month‐old male infant was brought to the outpatient clinic with a 3‐day history of fever, a 2‐day history of cough, and decreased appetite for 10 days. He was delivered via cesarean section with a normal birth weight of 3200 g. According to the available birth history, he reportedly received 1 mg of vitamin K intramuscularly at birth, in accordance with the routine hospital protocol for neonates. The infant was predominantly formula‐fed, with occasional breastfeeding. There was no history of trauma, previous bleeding episodes, or known congenital disorders.

Clinical evaluation did not reveal jaundice, acholic stool, hepatomegaly, chronic diarrhea, or failure to thrive suggestive of hepatobiliary disease or severe malabsorption. However, advanced investigations for biliary atresia, cystic fibrosis, inherited metabolic disorders, PIVKA‐II, serum vitamin K level, and coagulation factor assays were not available in this resource‐limited diagnostic setting.

On examination at the outpatient clinic, the infant was pale and febrile, with a temperature of 38.7°C and a heart rate of 132 beats per minute. Laboratory investigations revealed a hemoglobin level of 9 g/dL and a white blood cell count of 16,500/μL. A provisional diagnosis of sepsis with anemia was made, and intramuscular ceftriaxone was initiated along with supportive treatment for anemia.

Shortly after the first intramuscular dose, the patient developed a hematoma at the left thigh injection site, accompanied by active bleeding (Figure 1). Further investigations revealed severe coagulopathy, including an international normalized ratio (INR) > 4, prothrombin time (PT) > 120 s, and activated partial thromboplastin time (aPTT) > 120 s. The hemoglobin level decreased to 6.3 g/dL, and the WBC count increased to 22,500/μL. The patient was admitted for close monitoring and evaluation of a possible bleeding disorder. Because of the markedly prolonged PT/aPTT, active bleeding after minor intramuscular trauma, absence of previous bleeding history, ICH, and rapid correction after parenteral vitamin K, late‐onset VKDB was strongly suspected clinically although alternative causes of coagulopathy could not be completely excluded. During hospitalization, hemoglobin levels continued to decline, and the infant subsequently developed seizures. Parenteral vitamin K was administered to control active bleeding at the injection site. Despite normalization of coagulation parameters, the patient developed persistent seizures and worsening consciousness, necessitating admission to the intensive care unit (ICU) for 5 days (Table 1). Neuroimaging revealed subdural hematoma with suspected mild mass effect, asymmetric lateral ventricles, and associated hypoxic–ischemic cerebral injury (Figure 2).

**FIGURE 1:**
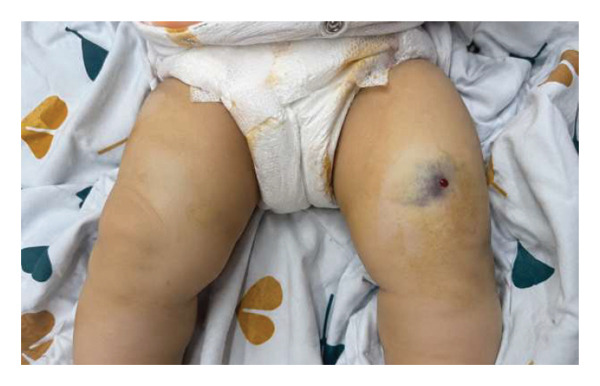
Left thigh injection‐site hematoma with active bleeding.

**TABLE 1:** Clinical timeline of the patient’s presentation, investigation, treatment, and outcome.

Timeline	Clinical course
Day‐10	Decreased appetite began.
Day‐3	Fever developed.
Day‐2	Cough appeared.
Day 0	Outpatient evaluation; anemia and leukocytosis detected; intramuscular ceftriaxone administered.
Same day	Injection‐site hematoma and active bleeding developed.
Admission	Severe coagulopathy identified (INR > 4, PT/aPTT > 120 s); hemoglobin decreased to 6.3 g/dL.
Hospital Day 1	Parenteral vitamin K administered; coagulation parameters improved.
Hospital Days 1‐2	Seizures and altered consciousness developed.
Hospital Day 2	ICU admission; neuroimaging revealed subdural hematoma with midline shift and hypoxic–ischemic injury.
ICU course	Received anticonvulsants, blood transfusion, antimicrobial therapy, and supportive care.
Hospital Day 10	Discharged in stable condition without apparent neurological deficits; follow‐up advised.

**FIGURE 2:**
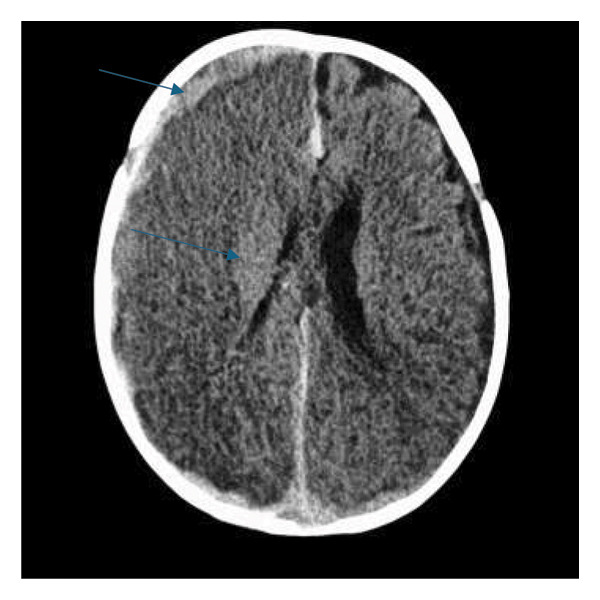
Head CT scan showing subdural hematoma with suspected mild mass effect, asymmetric lateral ventricles, and associated hypoxic–ischemic cerebral injury.

In the ICU, the patient received supportive therapy, including seizure control, blood transfusion for anemia correction, and treatment of sepsis. During hospitalization, seizures resolved, neurological function returned to baseline, and after a total hospital stay of 10 days, the patient was discharged in stable condition without apparent neurological deficits. Long‐term neurodevelopmental follow‐up was advised because delayed neurological sequelae may occur after ICH in infancy.

## 3. Discussion

VKDB remains a critical yet preventable cause of hemorrhagic complications in infants, particularly in its late‐onset form, which occurs from 2 weeks to 6 months of age. The present case from Herat, Afghanistan, is best interpreted as clinically probable late‐onset VKDB in a resource‐limited diagnostic setting. The clinical interest of this case is not that late VKDB after prophylaxis is entirely novel because such cases have been reported previously [8]. Rather, the importance lies in the unusual combination of reported neonatal prophylaxis, predominant formula feeding, injection‐site hematoma after intramuscular ceftriaxone, severe ICH, limited access to confirmatory testing, and favorable apparent neurological recovery despite serious neuroimaging findings.

Several clinical features supported probable VKDB in this infant: markedly prolonged PT and aPTT, active bleeding after minor intramuscular trauma, absence of previous bleeding history, subdural hematoma typical of late VKDB, and rapid correction of coagulation parameters after parenteral vitamin K. These findings are consistent with previous reports describing late VKDB as a cause of severe coagulopathy and ICH in young infants [1–4, 6, 7]. However, the diagnosis should be interpreted cautiously because PIVKA‐II measurement, serum vitamin K levels, coagulation factor assays, fibrinogen assessment, and genetic testing for inherited bleeding disorders were not available. Therefore, alternative inherited or acquired coagulopathies could not be completely excluded.

Potential secondary causes were considered clinically. The infant had no jaundice, acholic stool, hepatomegaly, chronic diarrhea, or failure to thrive, which made clinically evident hepatobiliary disease or severe malabsorption less likely. Nevertheless, advanced evaluation for biliary atresia, cystic fibrosis, inherited metabolic disease, or subtle liver dysfunction was not available. This uncertainty is important because hepatobiliary disorders have been associated with vitamin K deficiency and may also complicate the interpretation of subdural hemorrhage in infancy [12].

The occurrence of probable VKDB despite reported prophylaxis and predominant formula feeding requires cautious discussion. A single intramuscular dose at birth is highly effective and remains the standard preventive intervention; this report should not be interpreted as evidence against neonatal prophylaxis [9–11]. Several explanations remain possible but speculative in this case, including the incomplete documentation of prophylaxis, cold‐chain or storage limitations affecting vitamin K potency, variability in formula quality or fortification, reduced intake during illness, transient impairment of vitamin K absorption, or infection‐related effects on vitamin K metabolism. Because confirmatory testing was unavailable, these mechanisms cannot be proven from this case alone.

Intercurrent infection may have contributed to the clinical decompensation. The infant presented with fever, cough, leukocytosis, and suspected sepsis before the bleeding episode. Infection can reduce oral intake, disturb intestinal microbiota, and potentially impair hepatic synthesis or recycling of vitamin K‐dependent clotting factors. Antibiotic exposure may also alter gut flora although in this case, the injection primarily acted as a minor traumatic trigger that unmasked bleeding. Similar reports have highlighted that late VKDB may become clinically apparent after minor trauma or routine procedures when underlying coagulopathy is present [13, 14].

ICH is the most serious manifestation of late‐onset VKDB and has been reported in a high proportion of affected infants [2, 3, 6, 7]. In the present case, seizures and altered consciousness developed after the onset of injection‐site bleeding, and neuroimaging revealed subdural hematoma with suspected mild mass effect, asymmetric lateral ventricles, and associated hypoxic–ischemic cerebral injury. This clinical course parallels previous reports of infants presenting with seizures, altered consciousness, or acute neurological deterioration due to late VKDB‐related ICH [5, 15]. Prompt parenteral vitamin K, seizure control, blood transfusion, antimicrobial therapy, and intensive supportive care likely contributed to the favorable immediate outcome.

The absence of apparent neurological deficits at discharge should be interpreted with caution. Infants with ICH remain at risk for delayed neurodevelopmental impairment, epilepsy, motor deficits, and cognitive or behavioral sequelae, even when early clinical recovery appears complete [2, 4]. For this reason, long‐term neurodevelopmental follow‐up, seizure surveillance, and repeat neurological assessment are important components of postdischarge care. In resource‐limited settings, however, financial barriers, travel distance, and limited pediatric neurology services may make sustained follow‐up difficult.

The Afghan healthcare context is central to the interpretation of this case. Limited neonatal surveillance systems, restricted access to pediatric hematology and neurosurgical services, delayed neuroimaging availability, and financial barriers may delay diagnosis and treatment of VKDB. Advanced coagulation testing such as PIVKA‐II, factor assays, and genetic evaluation is often unavailable, forcing clinicians to rely on syndromic diagnosis and response to treatment. These limitations are relevant not only for this case but also for case reporting from similar resource‐limited settings, where diagnostic certainty may be lower than in tertiary centers with comprehensive laboratory support.

Management was complicated by significant resource limitations, including restricted access to advanced coagulation assays, pediatric hematology consultation, neurosurgical intervention, and long‐term neurodevelopmental follow‐up services. These limitations affected the ability to fully characterize the underlying mechanism of coagulopathy. They also highlight the importance of early clinical recognition, prompt vitamin K administration when VKDB is suspected, careful avoidance of unnecessary intramuscular injections in bleeding infants, and urgent neuroimaging when seizures or altered consciousness occur.

This report has several limitations. Advanced investigations including PIVKA‐II measurement, coagulation factor assays, vitamin K serum levels, fibrinogen studies, and genetic testing for inherited bleeding disorders were not available in our setting. Therefore, although the clinical presentation and rapid correction after vitamin K administration strongly supported probable VKDB, alternative causes of coagulopathy could not be completely excluded. In addition, the history of vitamin K prophylaxis and feeding practices depended on available documentation and parental report, and the long‐term neurological outcome remains uncertain without extended follow‐up.

From a public health perspective, the case supports strengthening—not questioning—vitamin K prophylaxis programs. Surveillance data show that VKDB is largely preventable when prophylaxis is reliably administered and completed [9–11]. In Afghanistan and similar settings, improved documentation of birth prophylaxis, parental education, clinical awareness of bleeding after minor procedures, and better access to coagulation testing and neuroimaging may reduce morbidity and improve diagnostic accuracy.

## 4. Conclusion

This case suggests probable late‐onset VKDB despite reported neonatal vitamin K prophylaxis and predominant formula feeding, while acknowledging that complete exclusion of alternative inherited or acquired coagulopathy was limited by resource constraints. It emphasizes the importance of maintaining clinical suspicion for VKDB in infants with unexpected bleeding after minor procedures or intramuscular injections, promptly administering parenteral vitamin K when clinically indicated, performing coagulation assessment and neuroimaging when neurological symptoms occur, and arranging long‐term neurodevelopmental follow‐up after ICH.

This case should not be interpreted as evidence against neonatal vitamin K prophylaxis. Rather, it reinforces the need for reliable prophylaxis documentation, parental education, improved surveillance, and better access to pediatric hematology, neuroimaging, and follow‐up services in Afghanistan and other resource‐limited settings.

**Nomenclature**
VKDB: Vitamin K deficiency bleeding
INR: International normalized ratio
PT: Prothrombin time
aPTT: Activated partial thromboplastin time
ICU: Intensive care unit
ICH: Intracranial hemorrhage

## Author Contributions

S.A.A.W., M.M., A.R., A.Q.R., and N.A.S. conceptualized the report. All authors contributed to the clinical management and/or data acquisition, literature review, and drafting and critical revision of the manuscript.

## Funding

This study did not receive any specific grant from funding agencies in the public, commercial, or not‐for‐profit sectors.

## Disclosure

All authors have read and approved the final manuscript.

## Ethics Statement

Ethical approval for this case report was obtained from the Ethics Committee of Jami University (approval code: J.2026.1.18), Herat, Afghanistan. The study was conducted in accordance with the principles of the Declaration of Helsinki. Written informed consent was obtained from the patient’s parent/legal guardian prior to inclusion, with assurances regarding confidentiality and exclusive research use of the information.

## Consent

Written informed consent was obtained from the patient’s parent for publication of this case report and any accompanying images. A copy of the written consent is available for review by the Editor‐in‐Chief of this journal upon request.

## Conflicts of Interest

The authors declare no conflicts of interest.

## Patient Perspective

The parents described significant emotional distress during the period of seizures and ICU admission but expressed gratitude for the child’s recovery and the supportive care received during hospitalization.

## Supporting Information

Additional supporting information can be found online in the Supporting Information section.

## Supporting information


**Supporting Information** CARE_checklist_filled_for_submission.

## Data Availability

The datasets generated and/or analyzed during the current study are available from the corresponding author upon reasonable request.
