# Supplementary material for: Severe Late‐Onset Vitamin K Deficiency Bleeding Presenting With Subdural Hematoma and Seizures in a 4‐Month‐Old Infant: A Case Report From a Resource‐Limited Setting
Source: Case Rep Pediatr. 2026 Jun 30;2026:9370038. doi: 10.1155/crpe/9370038 (PMC13318902; doi:10.1155/crpe/9370038)
Supplement: Supplementary file 1 — Supporting Information CARE_checklist_filled_for_submission. [file CRPE-2026-9370038-s001.docx]

**CARE Checklist with Page and Line Numbers**

*Manuscript: Severe Late-Onset Vitamin K Deficiency Bleeding Presenting with Subdural Hematoma and Seizures in a 4-Month-Old Infant: A Case Report*

**Note:** Page and line numbers were matched directly from the manuscript version containing visible page numbering and line numbering. Items marked “Partially reported” or “Not reported” may need revision before submission.

| **Topic** | **Item** | **Checklist item description** | **Status** | **Reported on page/line** |
| --- | --- | --- | --- | --- |
| Title | 1 | The diagnosis or intervention of primary focus followed by the words “case report”. | Reported | Page 1, lines 1-4 |
| Key words | 2 | 2 to 5 key words that identify diagnoses or interventions in this case report, including “case report”. | Partially reported | Page 2, lines 31-32 (keywords provided, but “case report” is not listed and may need to be added) |
| Abstract | 3a | Introduction: What is unique about this case and what does it add to the scientific literature? | Reported | Page 1, lines 6-11; Page 2, lines 26-30 |
| Abstract | 3b | Main symptoms and/or important clinical findings. | Reported | Page 1, lines 12-20; Page 2, lines 21-25 |
| Abstract | 3c | The main diagnoses, therapeutic interventions, and outcomes. | Reported | Page 1, lines 18-20; Page 2, lines 21-25 |
| Abstract | 3d | Conclusion - What is the main take-away lesson from this case? | Reported | Page 2, lines 26-30 |
| Introduction | 4 | One or two paragraphs summarizing why this case is unique (may include references). | Reported | Page 2, lines 34-42; Page 3, lines 43-63; Page 4, lines 64-70 |
| Patient Information | 5a | De-identified patient specific information. | Reported | Page 4, lines 72-77 |
| Patient Information | 5b | Primary concerns and symptoms of the patient. | Reported | Page 4, lines 72-73 |
| Patient Information | 5c | Medical, family, and psycho-social history including relevant genetic information. | Partially reported | Page 4, lines 76-77 (bleeding/trauma/congenital history noted; family, psychosocial, and genetic history not specifically detailed) |
| Patient Information | 5d | Relevant past interventions with outcomes. | Reported | Page 4, lines 74-75 (vitamin K at birth) |
| Clinical Findings | 6 | Describe significant physical examination and important clinical findings. | Reported | Page 4, lines 78-80; Page 4, lines 83-86; Page 5, lines 87-94 |
| Timeline | 7 | Historical and current information from this episode of care organized as a timeline. | Partially reported | Page 4, lines 72-98 (chronology described in narrative form; no separate timeline table/figure included) |
| Diagnostic Assessment | 8a | Diagnostic testing (such as PE, laboratory testing, imaging, surveys). | Reported | Page 4, lines 78-80; Page 4, lines 83-86; Page 5, lines 87-94 |
| Diagnostic Assessment | 8b | Diagnostic challenges (such as access to testing, financial, or cultural). | Not reported | Not stated in the manuscript |
| Diagnostic Assessment | 8c | Diagnosis (including other diagnoses considered). | Reported | Page 4, lines 80-81; Page 5, lines 87-94 |
| Diagnostic Assessment | 8d | Prognosis (such as staging in oncology) where applicable. | Not applicable / partially reported | Page 5, lines 95-98; Page 8, lines 167-170 (clinical outcome described, but no formal prognosis section) |
| Therapeutic Intervention | 9a | Types of therapeutic intervention (such as pharmacologic, surgical, preventive, self-care). | Reported | Page 4, lines 81-82; Page 5, lines 90-98 |
| Therapeutic Intervention | 9b | Administration of therapeutic intervention (such as dosage, strength, duration). | Partially reported | Page 4, lines 74-75; Page 4, lines 81-82; Page 5, lines 90-98 (interventions named, but most treatment doses/duration are not fully detailed) |
| Therapeutic Intervention | 9c | Changes in therapeutic intervention (with rationale). | Partially reported | Page 5, lines 90-98 (escalation to ICU care and supportive therapy described, but rationale/details could be expanded) |
| Follow-up and Outcomes | 10a | Clinician- and patient-assessed outcomes (if available). | Reported | Page 5, lines 95-98; Page 8, lines 167-170 |
| Follow-up and Outcomes | 10b | Important follow-up diagnostic and other test results. | Partially reported | Page 5, lines 91-94 (normalization of coagulation parameters and neuroimaging findings reported) |
| Follow-up and Outcomes | 10c | Intervention adherence and tolerability (How was this assessed?). | Not reported | Not stated in the manuscript |
| Follow-up and Outcomes | 10d | Adverse and unanticipated events. | Reported | Page 4, lines 83-86; Page 5, lines 87-94 |
| Discussion | 11a | A scientific discussion of the strengths and limitations associated with this case report. | Partially reported | Page 6, lines 125-130; Page 7, lines 133-166; Page 8, lines 167-178 (scientific discussion present, but explicit limitations are not clearly stated) |
| Discussion | 11b | Discussion of the relevant medical literature with references. | Reported | Page 6, lines 125-130; Page 7, lines 133-166 |
| Discussion | 11c | The scientific rationale for any conclusions (including assessment of possible causes). | Reported | Page 7, lines 133-166; Page 8, lines 172-178 |
| Discussion | 11d | The primary take-away lessons of this case report in a one-paragraph conclusion (without references). | Reported | Page 8, lines 172-178 |
| Patient Perspective | 12 | The patient should share their perspective in one to two paragraphs on the treatments they received. | Not reported | Not stated in the manuscript |
| Informed Consent | 13 | Did the patient give informed consent? | Reported | Page 9, lines 182-186 |

**Legend:** Reported = fully addressed in current manuscript. Partially reported = present but incomplete for CARE requirements. Not reported = missing from current manuscript.
